# Activation of SNAT1/SLC38A1 in human breast cancer: correlation with p-Akt overexpression

**DOI:** 10.1186/1471-2407-13-343

**Published:** 2013-07-12

**Authors:** Kuo Wang, Fang Cao, Wenzheng Fang, Yongwei Hu, Ying Chen, Houzhong Ding, Guanzhen Yu

**Affiliations:** 1 Department of Surgery, The Affiliated Kunshan First People’s Hospital, Jiangsu University, Kunshan 215300, Jiangsu Province, China; 2 Department of Medical Oncology, Changzheng Hospital, Shanghai 200070, China; 3 Department of Pathology, Changhai Hospital, Shanghai 200433, China

**Keywords:** Breast cancer, Tissue microarray, SNAT1/SLC38A1, p-Akt, Immunohistochemistry

## Abstract

**Background:**

SNAT1 is a subtype of the amino acid transport system A that has been implicated to play a potential role in cancer development and progression, yet its role in breast cancer remains unclear. In present study, we detected SNAT1 expression in breast cancers and explored its underlying mechanism in promoting breast carcinogenesis.

**Methods:**

RT-PCR and Western blotting were performed to analyze the transcription and protein levels of SNAT1 in breast cancer cell lines and fresh tissues. Tissue microarray blocks containing breast cancer specimens obtained from 210 patients were constructed. Expression of SNAT1 in these specimens was analyzed using immunohistochemical studies. SNAT1 was down-regulated by SNAT1-shRNA in breast cancer cells and the functional significance was measured.

**Results:**

SNAT1 was up-regulated in breast cancer cell lines and breast cancer tissues. Overexpression of SNAT1 was observed in 127 cases (60.5%). Expression of SNAT1 was significantly associated with tumor size, nodal metastasis, advanced disease stage, Ki-67, and ER status. Suppression of endogenous SNAT1 leads to cell growth inhibition, cell cycle arrest, and apoptosis of 4T1 cells and lowered the phosphorylation level of Akt. SNAT1 expression correlated significantly with p-Akt expression in human breast cancer samples.

**Conclusions:**

The cross-talk between Akt signaling and SNAT1 might play a critical role in the development and progression of breast cancer, providing an important molecular basis for novel diagnostic markers and new attractive targets in the treatment of breast cancer patients.

## Background

Breast cancer is the most frequently diagnosed cancer and the leading cause of cancer-related death among females worldwide [1]. Due to early detection, progress in treatment strategies and advances in our understanding of the molecular mechanisms of breast cancer, therapeutic effect increases and patients have longer survival duration. Unfortunately, global breast cancer incidence is increasing and most of these patients inevitably die of cancer recurrence and metastasis [2]. Therefore, it’s essential to unveil the underlying mechanism of tumor progression and develop effective therapeutic strategies. So far, several oncogenic kinase signaling pathways have been considered as potential targets for cancer treatment. Among these pathways, PI3K/Akt/mTOR signaling has been shown to regulate cell proliferation, growth, migration and energy metabolism [3-5]. Activation of Akt and its clinical value have been widely reported in human breast cancer [4-7]. Recently, researchers show that the amino acid carrier plays an important role in various cell life activities, including energy metabolism, detoxication,neutrotransmission and most importantly malignant transformation of mammal cell. L-type amino acid transporter 1 (LAT1), for example, was widely investigated in various human solid tumors and increased expression of LAT1 was shown to be associated with tumor size, advanced disease stages, and Ki-67 labeling index and consequently with poor patient outcome [8-10]. Given the importance of Akt pathway and amino acid transporters in nutrients and energy metabolism of tumor cells, we hypothesized that Akt activation might be associated with up-regulation of amino acid transporters [11].

Among these amino acid transporters, system A has been found to be overexpressed in human solid cancers, including glioma [12], hepatoceller carcinoma [13] and hilar cholangiocarcinoma [14]. System A amino acid transporter has three members: SNAT1, SNAT2, and SNAT4 (previously referred to as ATA1, ATA2, and ATA3, respectively), encoded by the SLC38 gene family (*Slc38a1*, *Slc38a2*, and *Slc38a4*) [15-17]. Among these three members, SNAT1 was significantly elevated in hepatocellular carcinoma and cholangiocarcinoma [13,14]. Knocking down endogenous SNAT1 inhibited cell proliferation of HepG2 cells [13]. Moreover, SNAT1 expression significantly correlated with tumor recurrence and poor outcome of patients with changiocarcinoma [14]. However, the expression pattern of SNAT1 and its role in breast cancer development has not been fully demonstrated.

In the present study, we sought to determine the expression profiles of SNAT1 in breast cancers and cells and to investigate its correlation with p-Akt. *In vitro*, we further confirmed the association between SNAT1 expression and Akt activation, which controlled cell viability and colony formation.

## Methods

### Materials

Recombinant murine EGF was purchased from PeproTech Inc. (Rocky Hill, NJ). phospho-Akt (Ser473) antibody was purchased from Cell Signaling Technology (Beverly, MA). Anti-SLC38A1 antibody was from Abcam Company (Cambridge, UK). ß-actin and Ki-67 antibodies were from Santa Cruz Biotechnology (Santa Cruz, CA).

### Cell lines and culture conditions

The breast cancer cell lines MCF-7, MDA-MB-231 and 4T1 were purchased from the Cell Center of Chinese Academy of Sciences, Shanghai, China. MCF-7, MDA-MB-231 and 4T1 were maintained in DMEM with 10% fetal bovine serum (FBS) (Invitrogen Corp., Grand Island, NY). The cell lines were cultured in a 37°C humidified atmosphere containing 95% air and 5% CO_2_.

### Tissue samples and tissue microarray construction

Seventy patients with breast cancer from the Affiliated Kunshan First People’s Hospital, Jiangsu Province, China from 2007 to 2011 and 140 cases with breast cancer from the Department of Oncology, Changzheng Hospital, Shanghai, China from 2008–2011 were enrolled in this study. Hematoxylin and eosin (HE) stained slides were prepared and reviewed by two pathologists (Y.C. and G.Y.) to ensure the quality of tissue blocks. The patients’ medical records were reviewed to obtain data, including age at diagnosis, tumor size, nodal metastases, and disease stage. These patient characteristics are listed in Table 1. All of these patients received no preoperative treatment, either radiotherapy or chemotherapy.

**Table 1:** Association between SNAT1 and p-Akt expression and clinicopathologic factors in breast cancer

**Clinicopathological variables**		**N (%)**	**SNAT1 (%)**		***P***	**p-Akt (%)**		***P***
			**Positive**	**Negative**		**Positive**	**Negative**	
Age(y)								
	<50 y	96(45.7)	58(60.4)	38(39.6)	0.987	64(66.7)	32(33.3)	0.509
	≥50 y	114(54.3)	69(60.5)	45(39.5)		71(62.3)	43(37.7)	
pT								
	pT1/2	174(82.9)	93(53.4)	81(46.6)	<0.001	104(59.8)	70(40.2)	0.003
	pT3/4	36(17.1)	34(94.4)	2(5.6)		31(86.1)	5(13.9)	
pN								
	No	136(64.8)	58(42.6)	78(57.4)	<0.001	64(47.1)	72(52.9)	<0.001
	Yes	74(35.2)	69(93.2)	5(6.8)		71(95.9)	3(4.1)	
Disease stage								
	I/II	168(80.0)	85(50.6)	83(49.4)	<0.001	96(57.1)	72(42.9)	<0.001
	III/IV	42(20.0)	42(97.6)	1(2.4)		39(92.9)	3(7.1)	
Her2								
	+	53(25.2)	32(60.4)	21(39.6)	0.986	37(69.8)	16(30.2)	0.332
	-	157(74.8)	95(60.5)	62(39.5)		98(62.4)	59(37.6)	
Ki67								
	+	166(79.0)	109(65.7)	57(34.3)	0.003	112(67.5)	54(32.5)	0.061
	-	44(21.0)	18(40.9)	26(59.1)		23(52.3)	21(47.7)	
ER								
	+	117(55.7)	60(47.2)	57(48.7)	0.002	67(57.3)	50(42.7)	0.017
	-	93(44.3)	67(72.0)	26(28.0)		68(73.1)	25(26.9)	
PR								
	+	105(50.0)	60(57.1)	45(42.9)	0.323	70(66.7)	35(33.3)	0.471
	-	105(50.0)	67(63.8)	38(36.2)		65(61.9)	40(38.1)	
Total		210	127(60.5)	83(39.5)		135(64.3)	75(35.7)	

Five paraffin-embedded tissue microarray blocks of normal and tumor tissue specimens obtained from these patients were created using a manual arrayer (Beecher Instruments, Sun Prairie, WI, USA). Forty-five cases had one 1.5-mm core of nonneoplastic tissue and two 1.5 mm cores of primary tumor tissues. The other cases only had two 1.5 mm cores of primary tumor tissue. Besides, four fresh breast cancer tissues and matched fresh nonneoplastic tissues were used to detect the expression levels of SNAT1 mRNA and protein. Ethical review committees (Institutional Review Board of the Affiliated Kunshan First People’s Hospital, Jiangsu University and Institutional Review Board of Changzheng Hospital, Shanghai) approved the use of all tissues and clinical information (KS2008-01 and CZEC2001-01).

### RNA preparation and reverse transcription-polymerase chain reaction

Total RNA was isolated from breast cancer cell lines and homogenised breast cancer samples using the AB gene Total RNA Isolation Reagent (Advanced Biotechnologies Ltd., Epsom, Surrey, UK). RNA concentration and quality were determined by spectrophotometric measurement (WPA UV 1101, Biotech Photometer, Cambridge, UK). cDNA was generated from 1 ug of each RNA sample and a reverse transcribed using a transcription kit (Takara, Kyoto, Japan). mRNA levels of SNAT1were assessed using the specific oligonucleotide primer pairs SNAT1 (sense: CCAGTGGCCTAGCTGGTACCAC and antisense: TCCCCAGCGAAAGTTGACTCAGAC); As an internal control, we used the β-actin primers (sense: GCTGTCACCTTCACCGTTC and antisense: CCATCGTCCACCGCAAAT).

### Immunohistochemical analysis and evaluation of immunostaining

4 μm sections of paraffin-embedded tissue microarrays blocks were prepared and processed for SNAT1 (dilution 1:50, ab59721; Abcam, Cambridge, UK) and p-Akt (dilution 1:50, 736E11; CST, Beverly, MA) proteins staining. A S-p kit (KIT-9710; MAIXIN, Fuzhou, China) was used to visualize antibody binding on the slides. Counterstaining was performed with hematoxylin. All slices were evaluated without knowledge of the expression of another marker. SNAT1 and p-Akt protein expression in the 210 cases was evaluated by two individuals (C.Y. and G.Y.) under an Olympus CX31 microscope (Olympus, Center Valley, PA).

The expression of the proteins was evaluated using a semiquantitative scoring system. Staining was graded on a scale of 0–2 (0=negative staining [no staining of any tumor cells], 1=weakly expression [staining of <25% of tumor cells], and 2=high expression [staining of ≥25% of tumor cells]). Only a score of 2 was regarded as overexpression [18]. Staining was scored independently by two individuals who were blinded to each other’s findings.

### Western blot analysis

Breast cancer cell lines, breast cancer specimens and matched non-tumor tissues were prepared for Western blot analyses. Standard Western blotting was performed using a rabbit antibody against human SNAT1 (1:1000) and p-Akt (1:1000) and an anti-rabbit IgG antibody, which was a horseradish peroxidaselinked F(ab’)_2_ fragments obtained from a donkey (Amersham). Equal protein sample loading was monitored by probing the same membrane filter with an anti-β-actin antibody.

### Plasmids and transfections

The shRNA-SNAT1 and unspecific scrambled shRNA plasmids were purchased from Genechem Company, Shanghai, China. At 24 hours before transfection, 1×10^5^ cells were seeded in six well plates. Transfection of shRNA was carried out using Lipofectamine™ 2000 reagent (Invitrogen, Karlsruhe, Germany) and 4 ng shRNA plasmid per well according to the manufacturer’s instructions.

### Cell proliferation assay

At 12 hours after transfection, cells were digested and 5000 cells were seeded in 96-well plates and incubated in medium with 10% FBS. At 24 h, 48 h, and 72 h, CCK8 assay (Dojindo Kumamoto, Japan) was performed to measure the final results. The experiment was repeated three times independently.

### Colony formation assay

At 24 hours after transfection, cells were digested and seeded in 6-well plates in triplicate at a density of 500 cells/well for 14 days at 37°C. The colonies were fixed with methanol/acetone (1:1) and stained with crystal violet. Colonies with cell numbers of more than 50 cells per colony were counted.

### Flow cytometric analysis

Flow cytometric analysis was performed as described previously to determine the effects of SNAT1-shRNA on cell cycle distribution and apoptosis [19,20]. Briefly, 4T1 cells, grown in 6-well plates (2 × 10^5^ cells/well), were synchronized at the G1/S boundary after starvation with basal medium for 24 hours, followed by transfection with SNAT-shRNA or shRNA vector for 48 hours. At the indicated time, cells were harvested by trypsinization and fixed with 70% ethanol, and measured following the manufacturer’s protocol (KEY GEN, Nanjing, China). Cell cycle distribution and apoptosis was analyzed by flow cytometry (FACSCalibur, BD Biosciences, Bedford, MA).

### Statistical analysis

Statistical analysis was performed using the SPSS 16.0 statistical software program for Microsoft Windows. Categorical data were analyzed using χ^2^ statistics tests. Within-group correlations of continuous and ordinal variables were assessed using Pearson’s R correlation coefficient or Spearman correlation coefficient when appropriate. The Kaplan-Meier method was used to estimate survival rates, and the log-rank test was used to assess survival differences between groups. The significance of the in vitro results was determined by using the Student t test (two tailed). Two-sided *P* value <0.05 was considered statistically significant.

## Results

### SNAT1 expression in patients with breast cancer

**Figure 1:**
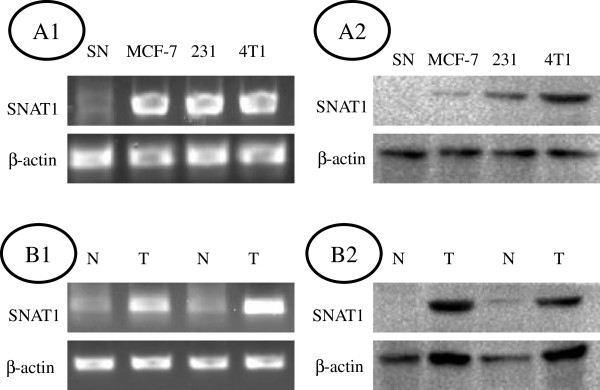
**Expression patterns of SNAT1 in breast cancer cell lines and human breast cancer specimens. (A1)** SNAT1 mRNA was overexpressed in MCF-7, MDA231, and 4T1 cells lines compared with normal breast tissues (SN); **(A2)** SNAT1 protein was overexpressed in MCF-7, MDA231, and 4T1 cells lines compared with normal breast tissues (SN); **(B1)** Overexpression of SNAT1 mRNA was observed in human breast cancer tissues (T) compared with that in matched noncancerous tissues (N); **(B2)** Overexpression of SNAT1 protein was seen in human breast cancer tissues (T) compared with that in matched noncancerous tissues (N).

To analyze the expression pattern of SNAT1 in breast cancer, we firstly examined its mRNA and protein levels in breast cancer cell lines and breast cancer specimens and matched non-tumor tissues. As shown in Figure 1 A1 and B1, the level of SNAT1 mRNA was highly expressed in cancer cell lines and cancers compared with non-cancer tissues. Similarly, SNAT1 protein levels were evaluated in breast cancer cell lines and cancers compared with non-cancer samples (Figure 1 A2 and B2). This result was further confirmed by immunohistochemistry.

**Figure 2:**
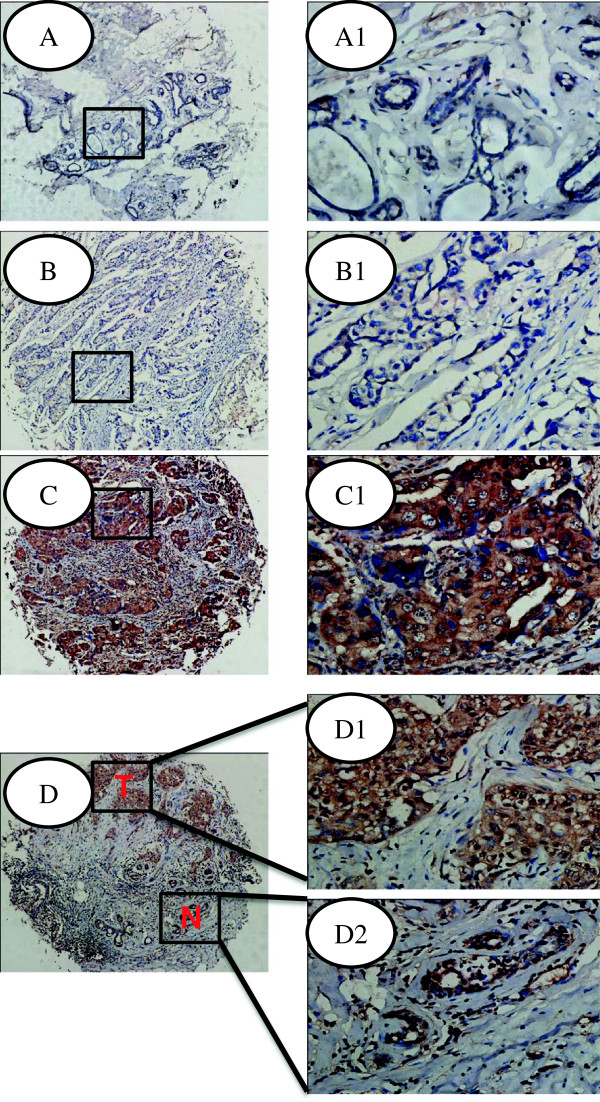
**Analysis of SNAT1 expression in human breast cancers and adjacent normal specimens. (A)** Normal (nonneoplastic) breast epithelium with negative expression of SNAT1; **(B)** Negative SNAT1 expression in breast cancer specimens; **(C)** Representative SNAT1 positive expression in breast cancer specimens; **(D)** High level of SNAT1 expression in tumor cells (T) and low SNAT1 expression in nonneoplastic breast epithelium (N); A1, B1, C1, D1, D2: Enlargement of tissues in the frames from **A**, **B**, **C**, **D**, respectively. Original magnification of **A**, **B**, **C**, **D**: 100×; Original magnification of A1, B1, C1, D1-2: 400×.

Immunostaining showed that SNAT1 positive staining was preferentially cytoplasm-localized. The epithelium in normal breast samples showed negative or weakly SNAT1 expression (Figure 2A). However, drastically increased SNAT1 expression was observed in the tumor cells (Figure 2C). Interestingly, SNAT1 expression was up-regulated in the tumor cells compared with the adjacent non-cancerous breast epithelium from the same sample (Figure 2D). Consistent with the mRNA data, this analysis showed that SNAT1 protein level in breast cancer was remarkably higher than that in normal adjacent epithelium.

### Correlation between SNAT1 expression and clinicopathologic characteristics of breast cancer

According to SNAT1 expression, the breast cancer patients were divided into two groups: SNAT1 negative expressers (n=83) and SNAT1 positive expressers (n=127). Table 1 summarized the correlation between SNAT1 overexpression and clinicopathological parameters in breast cancer. No significant relationship was found between SNAT1 expression and age, HER2, and PR expression. However, a statistically significant association was observed between SNAT1 expression and tumor size, lymph node metastasis, disease stage, Ki-67, and ER. Activation of SNAT1 occurred more frequently in large breast tumors (invasion to level T3-T4) (94.4%) than in small ones (level T1-T2) (53.4%; *P*<0.001) and more frequently in breast cancer with regional LN metastasis (93.2%) than in N0-stage tumors (42.6%; *P*<0.001). In regard to TNM stage, overexpression of SNAT1 was significantly associated with advanced disease stage: 97.6% at stage III /IV and 50.6% at stage I/II (*P*<0.001). Moreover, SNAT1 upregulation correlated significantly with Ki-67 overexpression (*P=*0.003) (Additional file 1: Figure S1) and ER-negative expression (*P=*0.002).

### Knockdown of SNAT1 by shRNA induces cell growth inhibition and apoptosis of breast cancer cells by blocking Akt phosphorylation

**Figure 3:**
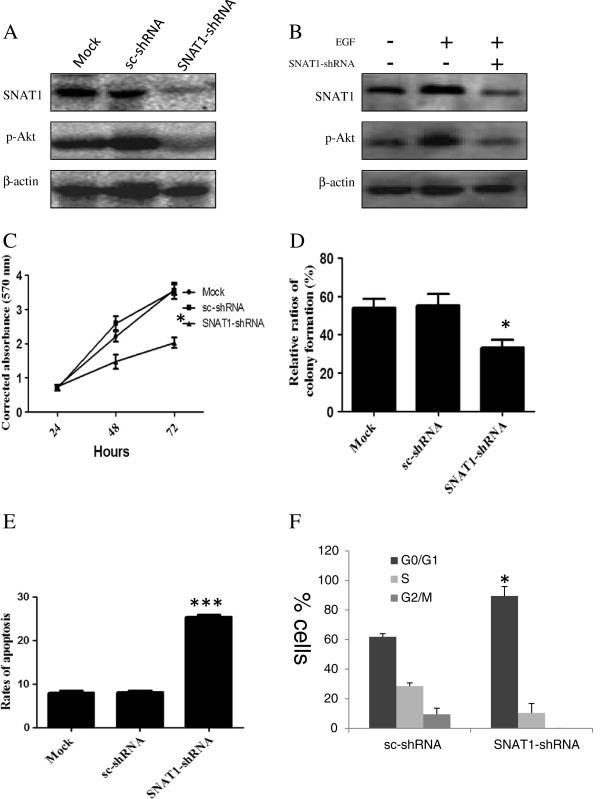
**Knockdown of SNAT1 induces cell growth inhibition, cell cycle arrested, and apoptosis of breast cancer cells by inhibiting phosphorylation of Akt. (A)** Western blot analysis of SNAT1 and p-Akt expression at 48 hours after the transfection of SNAT1-shRNA in 4T1 cells; **(B)** SNAT1-shRNA inhibits EGF induces p-Akt phosphorylation*.* 4T1 cells were transfected with SNAT1-shRNA with or without the presence of 50 ng/ml EGF. **(C)** 4T1 cells were transfected with SNAT1-shRNA or scrambled shRNA-transfected cells (sc-shRNA) at indicated times (24, 48, and 72 hours) and cell proliferation assay was performed; **(D)** 4T1 cells transfected with SNAT1-shRNA and sc-shRNA were grown in 6-well plates were incubated for 2 weeks. The numbers of the cell colonies were obtained and counted by 1-D gel quantity software QUANTITY ONE; **(E)** Cells were transfected with SNAT1-shRNA and sc-shRNA for 48 h. Then the cells were collected and the apoptosis rates were detected by flow cytometry; **(F)** After transfection for 48 h, 4T1 cells were harvested and cell cycle distributions were analyzed by flow cytometry. *P<0.05, ***P<0.001 considered statistically significant compared with sc-shRNA group.

**Figure 4:**
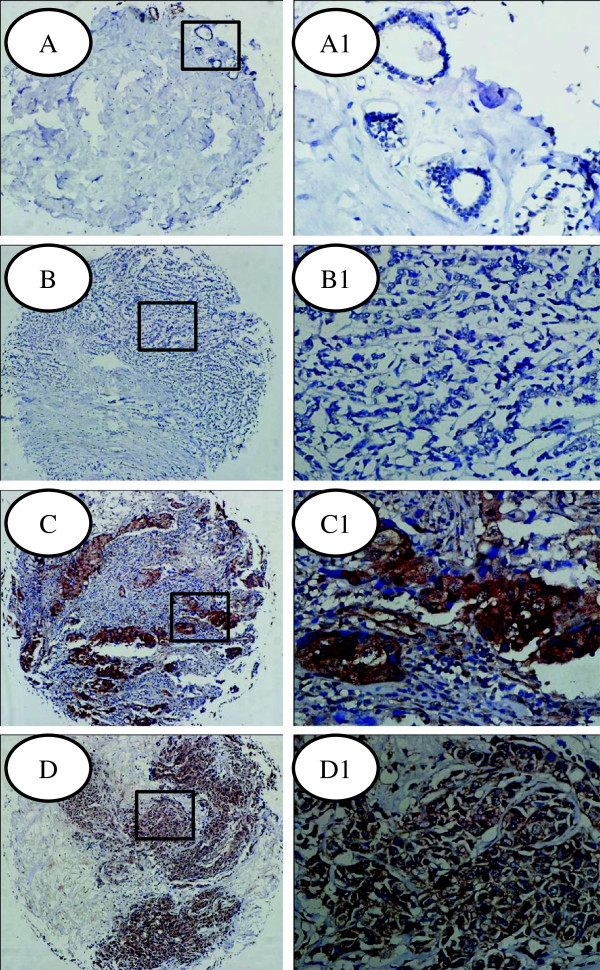
**Analysis of p-Akt expression in human breast cancers and adjacent normal specimens. (A)** Normal (nonneoplastic) breast epithelium; **(B)** Negative p-Akt expression in breast cancer specimens; **(C**, **D)** Representative p-Akt positive expression in breast cancer specimens; A1, B1, C1, D1: Enlargement of tissues in the frames from **A**, **B**, **C**, **D**, respectively. Original magnification of **A**, **B**, **C**, **D**: 100×; Original magnification of A1, B1, C1, D1-2: 400×.

Given the fact that SNAT1 expression was prominently activated in breast cancers, we further assessed the functional significance and the underlying mechanism of SNAT1 in breast cancer. As shown in Figure 3A, in 4T1 cells the transfection of SNAT1-shRNA results in a sharply loss of SNAT1 protein expression at 48 hours after the transfection. We also found that SNAT1 knockdown reduced the level of phosphorylation of Akt in 4T1 cells compared with the controls. When treated with 50 ng/ml of EGF, both SNAT1 and p-Akt protein levels increased, while the increase of p-Akt protein by EGF was partially reversed by SNAT1-shRNA (Figure 3B). The knockdown of SNAT1 significantly inhibited cell viability (Figure 3C) as well as colony formation (Figure 3D) of 4T1 cells. Meanwhile, SNAT1-downregulation leaded to cell cycle arrested at G0/G1 and increased apoptosis of 4T1 cells compared with shRNA empty vector transfected breast cancer cells (Figure 3E, F). These results suggest that the inhibitory effect of SNAT1-shRNA on 4T1 cells occurs partially through blocking Akt phosphorylation.

p-Akt immunostaining was of cytoplasm- or nuclear-localized. Negative or weakly expression of p-Akt was found in normal breast samples (Figure 4A), while increased expression of p-Akt was observed in 64.3% (135/210) cases. As shown in Table 1, a significant association was observed between p-Akt expression and tumor size, lymph node metastasis, advanced disease stage, and ER negative expression. There was no significant relationship between p-Akt expression and age, HER, Ki67, and PR status.

### Co-expression of p-Akt and SNAT1 in breast cancer specimens

**Figure 5:**
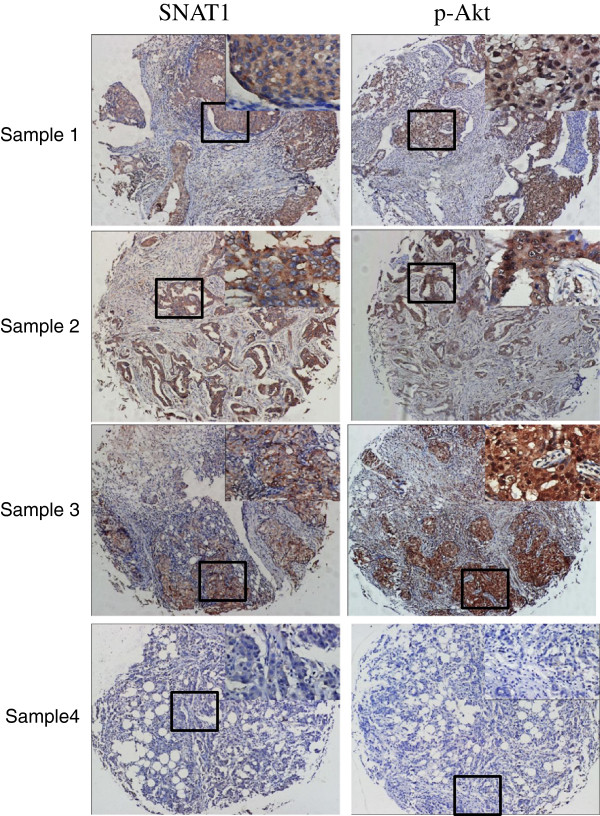
**Representative pictures showing co-expression of SNAT1 and p-Akt in human breast cancers from the same patients.** Original magnification of the big pictures: 100×; Original magnification of the small pictures: 400×.

Table 2 presented that SNAT1 expression significantly correlated with p-Akt expression (r=0.780, *P*<0.001). Co-expression of p-Akt and SNAT1 were observed in 120 (57.1%) tumors, while 68 (32.4%) tumors showed no expression of both. As shown in Figure 5, SNAT1 expression co-localized with p-Akt expression in the same specimens.

**Table 2:** Correlation between SNAT1 and p-Akt expression in breast cancer

		**SNAT1**		***P***	**r**
		**-**	**+**		
p-Akt	-	68	7	<0.001	0.780
	+	15	120		

### Overexpression of SNAT1 and p-Akt on survival in patients with breast cancer

**Figure 6:**
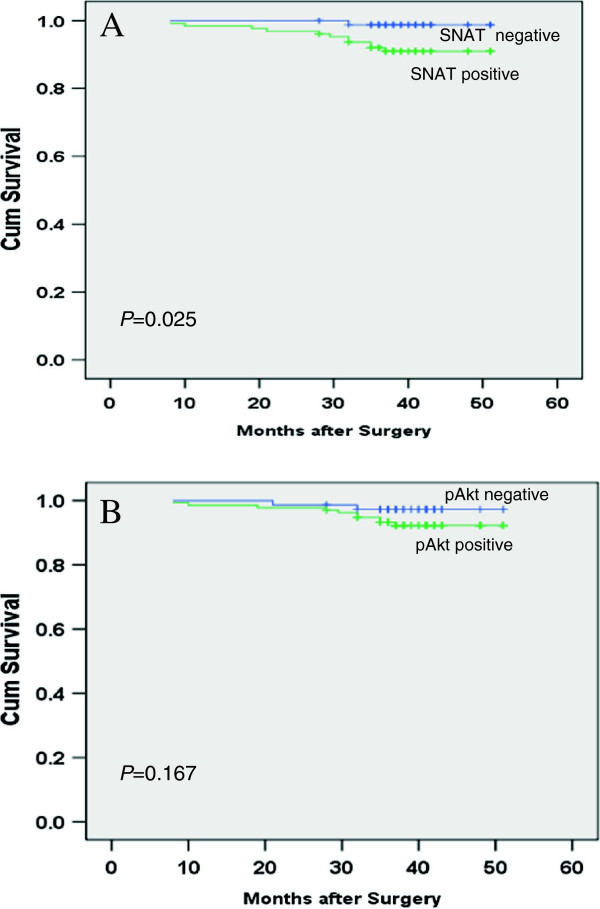
**Prognostic value of SNAT1 and p-Akt expression in patients with breast cancer. (A)** Survival durations were significantly worse in patients with positive expression of SNAT1 (median survival, 48.8 mo) than in those with negative expression of SNAT1 (median survival, 50.8 mo; *P*= 0.025). **(B)** No significant difference of survival durations were found between patients with positive expression of p-Akt (median survival, 49.1 mo) and those with negative expression of p-Akt (median survival, 50.3 mo; *P*= 0.167).

The cohort consisted of 210 female patients with a median age of 49 years (range, 28–81 years). Clinical follow-up results were available for these patients (median follow-up duration, 38 months; range, 19–51 months). To the end of follow-up, only 12 of the 210 cases died of cancer. Of the 12 patients, 11 showed overexpression of SNAT1 and 10 showed overexpression of p-Akt. Patients with SNAT1 overexpression tumors had a significantly shorter median survival duration (48.8 months) than patients without SNAT1 overexpression tumors (50.8 months) (*P* = 0.025). Patients with p-Akt overexpression tumors had shorter median survival duration (49.1 months) compared with those without p-Akt overexpression tumors (50.3 months) (*P*=0.167; Figure 6).

## Discussion

In this study, we found that up-regulation of SNAT1 was significantly associated with (1) tumor size, lymph node metastasis and advanced disease stage, the most important clinical determinants of treatment and prognosis for breast cancer; (2) Ki-67 overexpression and negative ER expression, the most important biomarker guiding treatment and outcome for breast cancer; (3) elevated activity of Akt, determined by the expression of phosphorylated Akt (p-Akt). Moreover, knockdown of SNAT1 blocked phosphorylation of Akt and hence attenuated cell growth and induced apoptosis of human breast cancer 4T1 cells. Therefore, we provided novel molecular evidence that activation of SNAT1/Akt signaling may play a critical role in breast cancer development and progression.

Glutamine has various important functions in mammalian cells and glutamine transport across cell membranes has been extensively studied physiologically [21]. The glutamine transporter (ATA1/SNAT1/SAT1/SLC38A1) is a member of the system A transporter superfamily, providing metabolic fuel or precursors for glutathione synthesis [22]. Physiologically, this carrier is mainly distributed in placenta and brain tissues [21,23]. Recently, researchers revealed overexpression of SNAT1 in human solid malignant tumors, including hepatic carcinoma and changiocarcinoma [13,14]. In the present study, SNAT1 expression was increased in breast cancer cells and tumor specimens compared with normal tissues at both mRNA and protein levels, suggesting oncogenetic role of SNAT1 in breast carcinogenesis. This result was further confirmed by immunostaining, which revealed a higher expression of SNAT1 in 60.5% cancer specimens and a lower expression of SNAT1 in 11.1% paraneoplastic tissues. Meanwhile, SNAT1 overexpression is closely correlated to tumor size, lymph node metastasis, disease stage, Ki-67, and ER-negative expression, indicating that SNAT1 is particularly important to breast cancer progression. Interestingly, patients with SNAT1 overexpression had a poor outcome than those without SNAT1 overexpression, further supporting the potential role of SNAT1 in cancer development and suggesting SNAT1 as a good target for cancer therapy. However, the relatively short follow-up duration limited the exploration of SNAT1 as an independent predictor of survival of breast cancer.

We then discovered that the knockdown of SNAT1 by specific shRNA reduced the viability of 4T1 cells. This inhibition might due to cell cycle arrested and apoptosis induced by downregulating SNAT1. Our data are in line with a study showing that siRNA mediated suppression of endogenous *ATA1* lowered the viability of HepG2 cells [13]. Take together, these siRNA or shRNA experiments suggested the SNAT1 molecule is essential in maintaining tumor survival. It has been shown that maternal protein restriction in rat inhibits Akt/mTOR signaling and down-regulates SNAT1 protein expression [24]. In this study, p-Akt level was downregulated after transfection with SNAT1-shRNA in 4T1 cells. In particular, this inhibition was also observed for EGF-induced increase of p-Akt. These results provided a molecular basis for cross-talk between Akt and SNAT1.

Activation of AKT in human cancers induces multiple downstream cascades to promote cell survival, tumor growth and progression. Deregulation of AKT signaling was widely found in variety of human cancers including breast cancer. Previous studies demonstrated that overexpression of p-Akt was found in 30%~80% of cases with breast cancers [4-7]. Similarly, our data showed that p-Akt overexpression was observed in 64.3% cases and correlated significantly with tumor size, lymph node metastasis, disease stage, and ER-negative expression. Interestingly, we found that p-Akt expression co-localized with SNAT1 expression in caner specimens from the same patients. SNAT1(+)/p-Akt(+) was predominantly found in 120 cases, while SNAT1(−)/p-Akt(−) was in 68 cases, accounting 89.5% of all cases. This notion is supported by a recent study showing that Akt/mTOR signaling pathways and amino acid transporter activity can be simultaneously down-regulated by chronic maternal infusion of full-length adiponectin in pregnant mice [25]. Other studies also revealed an interaction between other amino acid transporters (SLC36A1 and LAT1) and Akt signaling pathway [26,27]. Taken together, the cross-talk between Akt and SNAT1 might play a critical role in cell growth and tumor metastasis. However, whether decreased expression of p-Akt is a feedback of cell growth inhibition by knocking down SNAT1 or a direct downstream target of SNAT1 needs further investigation.

## Conclusions

In summary, SNAT1 was frequently activated in human breast cancer and its overactivation/overexpression was associated with advanced tumor stage and nodal metastasis. Additional in vitro study revealed that knockdown of SNAT1 inhibited cell growth inhibition, cell cycle arrested, and apoptosis of 4T1 cells by blocking the phosphorylation of Akt. The cross-talk between Akt signaling and SNAT1 provides an important molecular basis for novel diagnostic markers and new attractive targets in the treatment of breast cancer patients.

## Competing interests

We declare no conflicts of interest with any other person or units.

## Authors’ contributions

KW participated in the design of the study, carried out the mRNA expression of SNAT1 in breast cancer and cells, the immunohistochemistry of tissue microarrays and analyzed the data. FC and YH participated the immunohistochemistry analysis of SNAT1 and p-Akt in breast cancer patients and assisted the analysis of data. FC and WF performed the cell biology study and the Western Blotting test. YC and GY participated evaluation of immunostaining and assisted the collection of clinical data . DH and GY participated in its design and coordination, and supervised the study. GY drafted the manuscript. KW, FC and WF contributed equally to this work. All authors read and approved the final manuscript.

## Pre-publication history

The pre-publication history for this paper can be accessed here:

http://www.biomedcentral.com/1471-2407/13/343/prepub

## Supplementary Material

Additional file 1: Figure S1 A significant association between SNAT1 and Ki-67 was observed in breast cancer specimens. (A) Representative pictures showing co-expression of SNAT1 and Ki-67 in human breast cancers from the same patient. Original magnification: 200× (B) Statistics showed a significant correlation between SNAT1 and Ki-67 (r=0.206, *P*=0.003). Click here for file
