# Supplementary figures and images for: Activation of SNAT1/SLC38A1 in human breast cancer: correlation with p-Akt overexpression
Source: BMC Cancer. 2013 Jul 12;13:343. doi: 10.1186/1471-2407-13-343 (PMC3729721; doi:10.1186/1471-2407-13-343)

## Slide 1
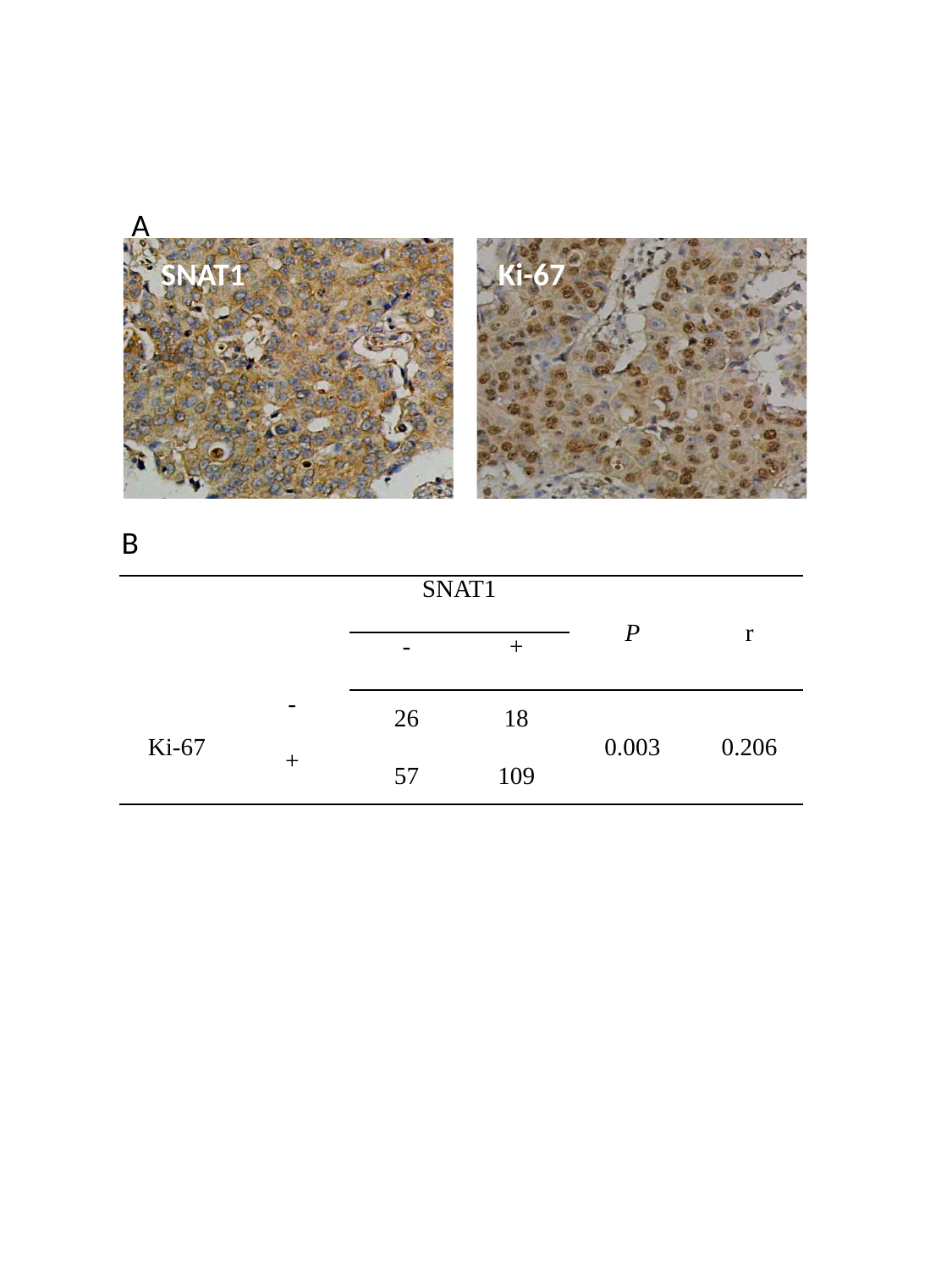

A
SNAT1
Ki-67
B
| | | SNAT1 | | P | r |
| --- | --- | --- | --- | --- | --- |
| | | - | + | | |
| Ki-67 | - | 26 | 18 | 0.003 | 0.206 |
| | + | 57 | 109 | | |

Supplement: Additional file 1: Figure S1 — A significant association between SNAT1 and Ki-67 was observed in breast cancer specimens. (A) Representative pictures showing co-expression of SNAT1 and Ki-67 in human breast cancers from the same patient. Original magnification: 200× (B) Statistics showed a significant correlation between SNAT1 and Ki-67 (r=0.206, P=0.003). [file 1471-2407-13-343-S1.pptx]
